# Skin Resistivity Value of Upper Trapezius Latent Trigger Points

**DOI:** 10.1155/2015/351726

**Published:** 2015-06-21

**Authors:** Elżbieta Skorupska, Jarosław Zawadziński, Agata Bednarek, Włodzimierz Samborski

**Affiliations:** ^1^Department of Rheumatology and Rehabilitation, Poznan University of Medical Sciences, 28 Czerwca 1958r., 60-545 Poznań, Poland; ^2^Department of Bionics and Bioimpedance, Poznan University of Medical Sciences, Parkowa 2, 60-775 Poznań, Poland

## Abstract

*Introduction*. The skin resistivity (SkR) measurement is commonly recommended for acupoints measurement, but for trigger points (TrPs) only one study is available. The purpose of the study was to evaluate SkR for latent TrPs compared to non-TrPs and the surrounding tissue. *Material and Methods*. Forty-two healthy volunteers with unilateral latent upper trapezius TrPs (12 men, 30 women) aged 21–23 (mean age: 22.1 ± 0.6 y) participated in the study. Keithley electrometer 610B was used for measuring SkR (Ag/AgCl self-adhesive, disposable ground electrode: 30 mm diameter). SkR was measured for latent TrPs and compared to opposite non-TrPs sites and the surrounding tissue. *Results*. The SkR decrease of TrPs-positive sites as compared to TrPs-negative sites and the surrounding tissue was confirmed. However, no statistically significant difference in the SkR value occurred when all data were analyzed. The same was confirmed after gender division and for TrPs-positive subjects examined for referred pain and twitch response presence. *Conclusion*. SkR reactive changes at latent TrPs are possible but the results were not consistent with the previous study. Thus, caution in applying SkR to latent TrPs isolation is recommended and its clinical use should not be encouraged yet. Further studies, especially on active TrPs, are yet required.

## 1. Introduction

Myofascial pain syndrome (MPS) is defined as a local pain syndrome characterized by (i) trigger points (TrPs), that is, limited sites of severe muscle tenderness or hypersensitivity, and (ii) a determined area of referred pain [1–3]. Trigger points are divided into latent and active, and the only differences between them are spontaneous pain characteristic of active points and the level of biochemicals. The incidence of TrPs is very common in general population, and the prevalence in around 30% of pain patients consulting in primary care has been proved [4].

The diagnosis of TrPs is based on essential clinical criteria (taut band, tender knot, pain recognition, and restricted range of motion) defined by Travell and Simons. Additionally, when one or more confirmatory signs such as referred pain, twitch response, or spontaneous electrical activity are confirmed, the diagnosis is more valid [3]. For research purposes, the most commonly tested muscle is the upper trapezius because of the high prevalence of TrPs in that muscle and easy access to the taut band [5, 6]. Moreover, Barbero et al. claimed that an experienced physiotherapist can reliably identify TrP locations in the upper trapezius muscle using a palpation protocol [7].

Over the last past years, a significant growth of interest in trigger points has been observed among researchers due to the objective confirmation of TrPs presence. The biopsy of the area defined as a trigger point has shown partial shortening and contraction of sarcomeres in particular muscle fibers, described as a “large, round, and dark muscle fiber.” This causes a statistically significant increase in the average myocyte diameter [8], confirmed recently by magnetic resonance elastography and ultrasonography [9–12]. Unfortunately, these techniques are not easily applicable to clinical practice at this time. That is why the diagnosis of TrPs is still based on palpatory diagnostic criteria and an easy and cheap method for TrPs confirmation is still required [3].

In the vicinity of TrPs, a deregulated motor end plate sustained by a neural loop of sensory and autonomic afferents in the central part of a TrP was confirmed. However, there is no overlap between TrPs and the innervation zone [13]. Additionally, Shah et al. [14] proved the presence of H+, BK, CGRP, SP, TNF-, IL-1, serotonin, and norepinephrine in active TrPs only. Electromyography has shown a spontaneous electrical activity (SEA) at TrPs during electrical silence of adjacent muscle fibers. SEA is defined as minute loci within TrPs that produce characteristic low-amplitude electrical activity [15, 16]. This indicates a direct relationship with bioelectric measurement and the possibility of using skin resistance as a noninvasive and easy TrPs measurement.

There is only one study that postulates the meaning of skin resistance used as a measurement tool for TrPs isolation from the surrounding tissue [17]. Moreover, based on that study, it seems useless to compare the value of TrPs skin resistivity to control group (healthy subjects). The average value of human resistance is 1500 ohms, but it varies greatly for different people and the results may be different even for one person due to many intrinsic and extrinsic factors. Because of this, the lateral presence of TrPs should be an inclusion criterion, with the opposite site corresponding to the common localization of TrPs becoming the control. Moreover, because it was proven that depending on gender skin resistance values for healthy subjects differ, skin resistivity should be investigated regarding gender [18].

The purpose of the study was to determine the SkR value of TrPs within the upper trapezius muscle compared to non-TrPs (control) and the surrounding tissue (norm). Additionally, the influence of gender differences and TrPs confirmatory signs on skin resistivity measurement was examined.

## 2. Material and Methods

### 2.1. Ethics Statement

The study protocol was approved by the Institutional Review Board of the Poznan University of Medical Sciences (number 790/12). The trial was registered in the Australian New Zealand Clinical Trials Registry: ACTRN12614001169639. Before their participation, all subjects were thoroughly informed of the methods and procedures used and gave their written consent to participate in this institutionally approved study carried out in accordance with the Declaration of Helsinki.

### 2.2. Patients

Forty-two healthy volunteers (12 men, 30 women) at the age of 21–23 (mean age: 22.1 ± 0.6 y) participated in the study. All volunteers participating in the trial filled in the clinical health questionnaire. The inclusion criteria were unilateral latent trigger points within the upper trapezius muscle. The exclusion criteria were (i) rheumatologic or neurological diseases and other serious medical conditions and real pain problems of the shoulder girdle and neck (because of the measurement methods of electrodermal skin resistance); (ii) surgery or/and posttraumatic incidence of the upper extremity and neck; (iii) diabetic problems; (iv) current use of some pain killers and other pharmacotherapies; (v) dermal problems of the upper back skin.

### 2.3. Trigger Points Confirmation

Before the main experiment, the participants were reexamined with respect to the presence of latent trigger points within the upper trapezius muscle (both left and right) defined by Travell and Simons as x1 and x2. The diagnosis was made by a clinician experienced in myofascial pain diagnosis according to Travell and Simons' criteria [3]. The confirmatory signs, namely, referred pain and twitch response, were examined. For the purposes of skin resistance measurement, each of the latent TrPs was marked and defined as TrPs-positive. The same was done for the area corresponding to the region common for TrPs with negative results named as non-TrPs (control). Then the examiner looked for four pain-free points on each side (not tender, without any features of TrPs) in the closest area to the previously marked crosses. When the four points were found, they were marked and named norm.

### 2.4. Skin Resistance Measurement

Experiments were performed with the subjects placed prone in a quiet room with the ambient temperature set at 23 ± 1°C. They rested for 15 min. before each trial.

**Figure 1:**
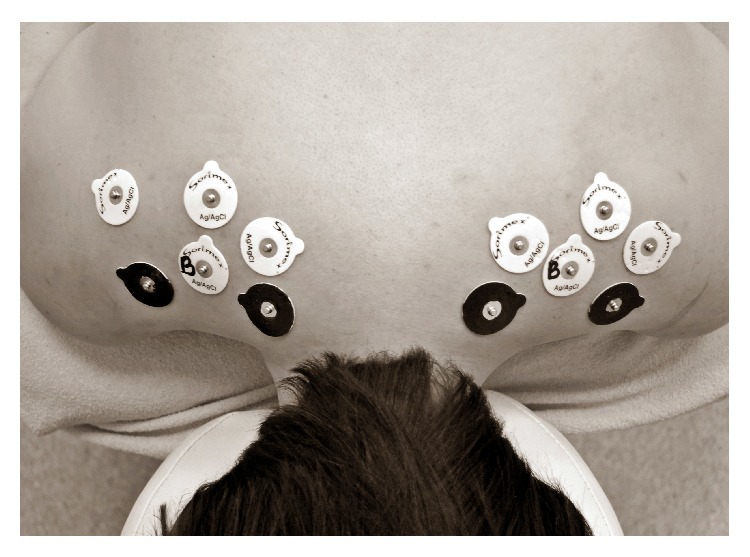
Example of TrPs, control (TrPs-negative), and norm electrodes localization during SkR measurement. The black electrodes show TrPs localization (x1 and x2) and, on the opposite site, next two black electrodes TrPs negative x1 and x2 localization. The electrodes with the letter “B” show “reference points,” and the three white electrodes on each side refer to the norm (not tender points of the surrounding tissue).

The participants were evaluated towards skin resistance in the marked area by an expert without any knowledge about TrPs examination results. During tests, a Keithley electrometer 610B was used for measuring skin resistance (Ag/AgCl self-adhesive, disposable ground electrode, 30 mm diameter). The electrodes were placed on the specific points of the skin. Two electrodes were put on the points defined as x1 and x2 TrPs within the upper trapezius (TrPs-positive and control) and four on other points showing no evidence of pathology (norm) (Figure 1). One of the norm points (positioned centrally in relation to the other) functioned as a reference point. Skin resistance (in ohms, [*Ω*]) was measured once in every marked point. The electrometer was rezeroed before every trail. Input parameters were turned off and the electrometer was corrected for internal noise to recalibrate as the participants changed. All tests were completed in a climate controlled room.

### 2.5. Data Analysis

Resistance values previously obtained from each of the marked points (TrPs-positive, control, and norm) were used to calculate the resistivity using the following formula:

(1) $$\begin{array}{c} \rho =\frac{RS}{x}, \end{array}$$

where *ρ* is resistivity [Ωm]; *R* is resistance [Ω]; *S* is electrode surface [m^2^]; *x* is distance between electrodes (reference and others) [m].

The mean value obtained from the calculations made for three norm points was determined in order to calculate the so-called personal norm for the pain-free area (without any TrPs features). Additionally, TrPs skin resistivity values were analyzed with regard to the gender and the presence of confirmatory signs (referred pain and twitch response).

### 2.6. Statistical Analysis

The two-tailed *t*-tests were performed in order to ensure that data are representative of the full population of possible data values. In order to compare the differences in skin resistivity values of TrPs-positive and TrPs-negative sites as compared to their control points, the two-tailed *t*-tests were applied. All of the above dependences with regard to gender division, referred pain, and twitch response presence were tested by the two-tailed *t*-test. Values in the text, figures, and tables are expressed as ± standard error of the mean (SEs). All levels of probability were set at a significant level of 0.05. The statistical analysis was performed using IBM SPSS Statistics, version 20.

## 3. Results

**Figure 2:**
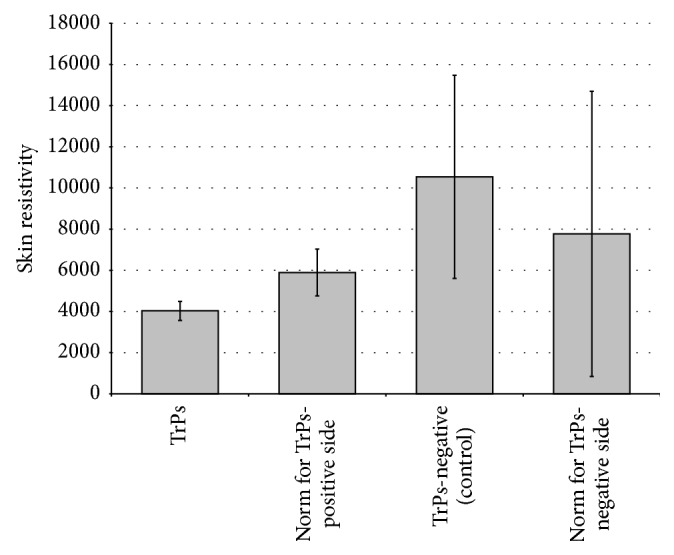
Mean value of skin resistivity for TrPs-positive and TrPs-negative compared to their norm.

The skin resistivity decrease of TrPs-positive sites compared to other measured sites, that is, TrPs-negative (control) and norm, was confirmed. However, the two-tailed *t*-tests showed no statistical significance for skin resistivity between TrPs-positive to norm (*p* = 0.59), TrPs-positive to TrPs-negative (control) (*p* = 0.19), and TrPs-negative (control) to norm (*p* = 0.12) when all data were analyzed.  Figure 2 presents the mean value of skin resistivity for TrPs-positive and TrPs-negative compared to their norm. After rejecting markedly different values, the same tendency was confirmed.

**Figure 3:**
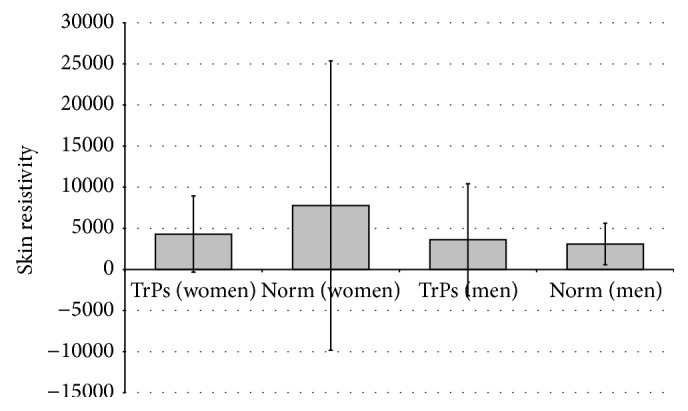
Mean value of skin resistivity for TrPs-positive compared to the surrounding tissue (norm) after sex division.

After sex division, a contrary tendency of skin resistivity value was observed: among women, SkR decrease of TrPs sites compared to the surrounding tissue (norm), and for men, SkR increase for TrPs compared to norm (Figure 3). For both subgroups, the two-tailed *t*-tests confirmed no significant changes in skin resistivity between TrPs-positive to the surrounding tissue (norm) (women *p* = 0.07; men *p* = 0.56) and TrPs-negative (control) (women *p* = 0.23; men *p* = 0.34) after sex division. After rejecting markedly different values, the same tendency was confirmed.

### 3.1. Skin Resistivity of TrPs Depending on Presence of Confirmatory Signs

**Figure 4:**
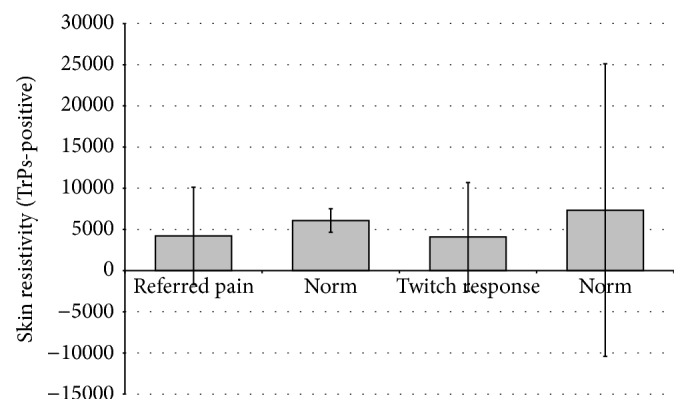
Mean value of TrPs skin resistivity dependent on the confirmatory sign presence.

For TrPs-positive subjects, skin resistivity values were analyzed with regard to referred pain and twitch response occurrence. There was no statistical difference between TrPs-positive sites with referred pain as compared to their norm (*p* = 0.17) and with regard to the presence of twitch response compared to their norm (*p* = 0.11) (Figure 4). The same was confirmed after sex division for referred pain: men: 3917.9 ± 7367.0 versus 2915.7 ± 1953.9 *Ω∗*m (*p* = 0.31); women: 4404.4 ± 4800.1 versus 8091.7 ± 18413.2 *Ω∗*m (*p* = 0.08). When twitch response presence was analyzed, for men it is 3335.2 ± 7729.7 versus 3483.8 ± 2861.7 *Ω∗*m (*p* = 0.91); women 4613.1 ± 5684.6 versus 10067.7 ± 22783.5 *Ω∗*m (*p* = 0.10). Confirmatory signs, namely, twitch response and referred pain, do not significantly differentiate skin resistivity of TrPs-positive with/without confirmatory signs.

## 4. Discussion

The main result of the present study is that skin resistivity (SkR) measurement does not significantly differentiate latent trigger point localization from the surrounding tissue and asymptomatic region corresponding to the common localization of TrPs (control) (Figure 2). This is contrary to the study by Shultz et al. [17] who recommended skin resistance measurement for trigger points (TrPs) confirmation. However, a similar tendency of the SkR value at TrPs site, namely, SkR decrease, compared to the surrounding tissue, was confirmed in both studies. That tendency seems valid for future studies because skin resistance decrease in conditions related to the sympathetic nervous system (SNS) activation was suggested [19, 20]. Interestingly, the latest data indicate the meaning of SNS in TrPs pain propagation [21–25]. However, it is not known whether SNS activity influences all TrPs or only severe active TrPs subjects. If we assume that only severe state of active TrPs is SNS-related, then only such cases can be SkR sensitive. However, no objective method of active TrPs division exists and, thus, it remains just an assumption.

Additionally, the distribution of standard deviations in the present study indicates that it is impossible to establish TrPs resistance norm range which could be later used in practical assessment of patients. There may be several reasons for this situation. Skin resistance value depends on many factors such as blood flow, thickness, and sweat glades activity. These factors can provoke constant changes during the day, which may consequently lead to skin resistance changes [26]. Thus, it is difficult to establish resistance norm for skin in vivo. Similar observations were proven for acupuncture studies where due to high variability the results were assessed as relative rather than absolute numbers [27].

Furthermore, the difference between the present and Shultz et al.'s [17] studies can be explained by a certain methodological issue. Some information within the discussion by Shultz et al. indicated diminished credibility. The authors stated that false-negative TrPs examination would make the difference between skin resistance values of non-TrPs and experimental group negligible [17]. However, trigger points diagnosis within the upper trapezius muscle is one of the easiest and, since reliable identification using a palpation protocol was proven, there should be no doubts regarding TrPs confirmation [7].

Additionally, the differences in results of undertaken studies may be explained through different methodologies that were applied to experiments. First of all, in the present study, AG/Cl electrodes were used in every measured point. Shultz et al. [17] used ultrasound gel and put a metal electrode to skin using the gel. The only ground electrode was Ag/Cl electrode. The great disadvantages of this method are differences in contact area (in the case of gel and electrode), as well as differences in electrode application pressure [28]. Secondly, we located a ground electrode in the center of the measurement area and tried to remain constant distance between measured points and the ground electrode. It is known that the location has significant influence on measurements because resistance depends on distance between electrodes [29]. Shultz et al. did not define the location of the ground electrode [17]. However, after the data presented is analyzed, it can be assumed that the ground electrode was located a few centimeters to the left from the measurement area. The methodological differences, including technical standards which influenced the study results, were indicated by others and are observed in the TrPs resistance study discussed above [30].

Another interesting aspect of the present study is data analysis after gender division. This was recommended in many studies because of the differences in skin thickness (greater in man) and skin blood flow (much greater in man), which influence skin as an electric conductor [18, 31, 32].

In the light of the present study that recommendation revealed interesting observation, data analysis after gender division in the present study showed a completely opposite reaction of SkR in the TrPs region, namely, a decrease or an increase depending on the sex (Figure 3). This situation may be explained by differences in sweat glands localization and mode of action; while the density of sweat glands in men is lower, in women sweating reaction is delayed and is less copious, which may influence electrodermal activity results [33].

Shultz et al. [17] explained their results and recommended the usefulness of TrPs skin resistance measurement basing on Shah's findings [14], which confirmed the existence of a localized hypoxic region in TrPs and a local increase in sensitizing substances in that area possibly inducing locally greater blood flow and secretion from sweat glands via stimulation of the autonomic nervous system. Shultz et al. [17] speculated that twitch response could increase the autonomic response which would cause changes to vasodilation and sweat secretion to the localized area. However, Shultz et al. did not analyze the meaning of twitch response presence, which was a differentiating factor in Shah's study [14]. The data in the present study confirmed that for TrPs localization there is no SkR difference dependent on the presence of twitch response or referred pain. However, maybe the SkR measurement just after needle-evoked twitch response could reveal some differences. Also perhaps it is not the twitch response presence but the severity of TrPs (active or latent form) that is important for the SkR outcome. Moreover, there are only two studies concerning SkR measurement, both based on the same muscle. Thus, other muscles with TrPs should be evaluated towards the SkR value compared to the surrounding tissue. Further studies concerning this idea are recommended in the future.

## 5. Conclusion

SkR reactive changes at latent TrPs are possible but the results were not consistent with the previous study. Thus, caution in applying SkR to latent TrPs isolation is recommended and its clinical use should not be encouraged yet. Further studies, especially on active TrPs, are yet required.
